# Ultraviolet-C as a Viable Reprocessing Method for Disposable Masks and Filtering Facepiece Respirators

**DOI:** 10.3390/polym13050801

**Published:** 2021-03-05

**Authors:** Talita Nicolau, Núbio Gomes Filho, Andrea Zille

**Affiliations:** 1 2C2T—Centre for Textile Science and Technology, University of Minho, 4800-058 Guimarães, Portugal; tali_nicolau@hotmail.com; 2 School of Economics and Management, University of Minho, 4710-57 Braga, Portugal; id7657@alunos.uminho.pt

**Keywords:** ultraviolet-C, surgical masks, filtering facepiece respirators, sterilization, germicidal capability, additional advantages, thermal deformation, shadowing, absorption effect, filtration power

## Abstract

In normal conditions, discarding single-use personal protective equipment after use is the rule for its users due to the possibility of being infected, particularly for masks and filtering facepiece respirators. When the demand for these protective tools is not satisfied by the companies supplying them, a scenario of shortages occurs, and new strategies must arise. One possible approach regards the disinfection of these pieces of equipment, but there are multiple methods. Analyzing these methods, Ultraviolet-C (UV-C) becomes an exciting option, given its germicidal capability. This paper aims to describe the state-of-the-art for UV-C sterilization in masks and filtering facepiece respirators. To achieve this goal, we adopted a systematic literature review in multiple databases added to a snowball method to make our sample as robust as possible and encompass a more significant number of studies. We found that UV-C’s germicidal capability is just as good as other sterilization methods. Combining this characteristic with other advantages makes UV-C sterilization desirable compared to other methods, despite its possible disadvantages.

## 1. Introduction

In normal conditions, single-use personal protective equipment (SUPPE) is discarded after use, once it might be infected [1]. However, when pandemics, such as the one of Coronavirus 2019 (COVID-19), happen, conditions for a perfect storm happen, creating a combination of two factors: (1) a higher demand-side pressure for those pieces of equipment on a global scale [1,2,3,4,5,6,7,8,9,10], added to (2) failure on the supply-side to meet these needs, as most SUPPE supply chains were in China, and they were unable to export their products [11]. When this perfect storm occurs, reprocessing SUPPE gains visibility as there is no universal treatment [10,12,13]. Despite the fact that there are now multiple vaccines, SUPPE demand will continue to suffer pressures until countries reach herd immunization, especially if countries start banning woven masks, as in Germany or France, due to virus mutations.

Reprocessing SUPPE is crucial because it tackles multiple problems, since (1) it gives a rapid solution to this shortage during a crisis (economic factor); (2) it reduces the environmental impact this increase in production would cause in terms of nonrecyclable waste (environmental facet); (3) it enables poorer countries to diminish their costs from highly inflated SUPPE at this moment (social aspect).

Facing this crisis, multiple organizations throughout the world (e.g., the F.D.A., the C.D.C., the E.C.D.C., the U.N.) have adjusted their infection control measures [14], issuing special authorizations for sterilizing SUPPE [15,16,17,18], as well as reports/guidelines about multiple reprocessing methods [19,20,21,22,23,24]. Even though such changes in perspective happened, there is still no universal option for reprocessing SUPPE. This lack of universal choice happens because each option may affect SUPPE’s efficiency and integrity, diminishing its barrier capability [1,4,7,8,10,14]. Given that there is no universal choice, reprocessing SUPPE must consider their materials, topologies, the resources at hand, and the markets’ acceptance [3,12,25]. The four major sterilization methods are thermal, chemical, radioactive, and energetic [3,8,12,13,26,27].

Thermal methods commonly deactivate microorganisms, such as viruses, given they denature their proteins [3,8]. However, these methods face two cardinal problems: they might deform SUPPE irreversibly and face scalability issues [3,8,9,12,13,14,20,26]. In order to deal with such problems, some researchers [28,29,30] use nanostructured materials with photothermal properties, such as poly(NIPAm-*co*-NIPMAm) hydrogels [28,29]. This characteristic enables the absorption of visible/near-infrared light, creating localized heat sources [28]. Although this solution might solve the previous problems, it is still in its infancy. Thus, the development of autophotothermal disinfection SUPPE [30] might prove useful in the future, but it lacks further research to evaluate its industrial feasibility.

The typical chemical methods used to sterilize SUPPE are Vaporized Hydrogen Peroxide (VH_2_O_2_) and Ethylene Oxide (EtO), and the first has been gaining the second’s market. Even though VH_2_O_2_ is ineffective on cellulose-based SUPPE, it is environmentally friendly, and EtO is carcinogenic [2,8,12,25]. The chemical methods have some cons, such as their residues causing allergic reactions and having an intense odor. They depend on specific machinery, and VH_2_O_2_ is highly unstable when vaporized, losing significant efficiency when condensed [3,5,7,9,12,25,31].

Regarding radioactive sterilization methods, the most common one uses gamma irradiation, and it is already primarily used for sterilizing medical tools on a large scale, but it is highly dependent on expensive machinery, it might cause irreversible deformities on SUPPE, and these methods use radioactive raw material [2,8,20,25,26].

Regarding the energetic methods, the water and the food industry already apply germicidal ultraviolet (UVGI) [2]. Ultraviolet C (UV-C), among the UVGI, can damage biological structures via the photodimerization process since both RNA and DNA bases strongly absorb UV-C [3]. UV-C is suitable due to its low cost, high throughput, ease of use, and no chemical residues left [6,12,32]. UV-C has some limitations related to SUPPE thermal deformation, shadowing, and absorption effects [4,5,8,27,31].

Considering this panorama, UV-C seems to be the method that is more suitable to tackle the problems (economic, environmental, and social) of selecting a sterilization method for SUPPE. Thus, this paper aims to describe the state-of-the-art for UV-C sterilization in masks and filtering facepiece respirators.

Among different SUPPE, masks and filtering facepiece respirators (FFRs) became necessary due to their primary role as a protective barrier from Coronavirus disease infection in the hospital and nonhospital environments [33]; therefore, this paper focuses on these SUPPE. This analysis allows observing if studies benefit from advantages and face the disadvantages described for UV-C and how they are overcoming them. Such evaluations are essential because UV-C is already useful in disinfecting other materials/equipment/products, but there remain doubts concerning its disinfection ability for masks/FFRs.

The authors have used a systematic literature review (SLR) on multiple databases to gather English-written publications that researched UV-C’s impacts on masks and FFRs. More information about the quantitative of analyzed studies and their sources (Table S1), SLR’s database description (Figure S1) and SLR’s database descriptive analysis (Figure S2) are available in Supplementary Materials.

## 2. UV-C Sterilization in Masks and Filtering Facepiece Respirators

This section compiles the SLR results describing the state-of-the-art for UV-C sterilization in masks and filtering facepiece respirators. Readers may find these studies’ samples, UV-C’s system setups, and results in Table 1, entitled SLR’s final database main features and results.

## 3. UV-C’s Germicidal Capability

Considering the studies in the SLR’s final database, 31 studies [1,6,34,35,36,37,38,39,40,41,42,43,44,45,46,47,49,50,52,55,56,57,58,59,60,61,63,64,65,66,85], representing 53.45% of the SLR database, attest to UV-C’s germicidal capability, as they find results of at least 3-log reduction using different biological indicators, conditions, and setups. These studies present results using UV-C as a single method of sterilization. In contrast, another five studies [44,48,51,66,74] use ultraviolet in hybrid models, four combined heat and UV-C, and one adds hydrogen peroxide. While two publications [48,74] do not evaluate UVGI alone, the other two [44,51] do, one attesting for it and another one not. The last one [66] simulates its efficiency. However, when the authors evaluate the hybrid model, they find reductions “well beyond 3” -log [51] (p. 13). Additionally, four research papers [1,47,55,56] indicate a relation between virucidal activity and the masks’ (or FFRs’) models. Summing these results up, all of the researchers tested for the germicidal capability of UVGI and found at least partial confirmation of it, being the majority working with UV-C and being in favor of its usage.

Besides its need to be germicidal for SUPPE, UV-C must have low cost, high throughput, ease of use, and reduce or leave behind no chemical byproducts [6,12,32]. The sum of these advantages creates appeal for this reprocessing method. These advantages can be either read solely for UVGI methods or in comparison with other (thermal, chemical, and radioactive) methods’ disadvantages.

## 4. UV-C’s Additional Advantages

Ultraviolet-C is normally regarded as a low-cost reprocessing method. From the SLR’s final sample, most publications used either adapted biosafety [6,49,50,57,58,59,61,83] or sterilization [33,56,86] cabinets; adapted chambers [7,39,43,48,64,66,75,78], rooms [51,68,69,85] or laminar flow cabinets [43,71,80,82]; the lamps alone [38,40,46,52,54,63,79,87] or tube racks [70]. Most of these resources are available in research departments or hospitals; making this method “reasonably (…) inexpensive” [7] (p. 515) or, at least, “a cost-effective alternative to heat or chemical decontamination” [69] (pp. 396–397).

Ten publications [34,37,42,44,45,60,62,73,76,77] adapted machines/robots or used specific UVGI cabinets. These options seemed more expensive approaches than the previous ones. This information does not completely invalidate the “low-cost” idea, as they might prove to be cost-effective once the facility is looking for reprocessing masks and might already own such devices, and they may be idle. Lastly, some researchers created prototypes [41,53], or built their own UV cabinets [1,35,55,65,67,72,74,84]. These self-built UV cabinets are sometimes built from scratch using inexpensive raw materials like aluminum [1,65], or they adapted other containers [55,67,72,74], such as metallic tool storage, an old freezer box, or a reflecting box.

Considering that UVGI methods are of high throughput, this advantage is not mentioned by every study. Assuming that most studies use small chambers [7,39,43,48,64,66,75,78], biosafety [6,50,57,58,59,61,83] or sterilization [33,56,86] cabinets, this could partially hinder this advantage, as SUPPE cannot be stacked (piled up) [47,76].

Although some publications [7,41,57] argue that reaching high throughput depends on adapting their systems’ setups, which increases their processes’ agility and consequently their throughput per round, finally, some studies [39,44,67,72,73] indicate an actual number of masks and FFRs disinfected per round. These numbers depend on the area each mask model has and the irradiated area the system has. Despite these studies, this capability becomes easily observed when researchers use adapted rooms [51,68,69,85] since they can disinfect multiple SUPPE at once.

In the matter of effortlessness application of UVGI, fewer studies [7,34,41,44,48,55,72,73,87] discuss it. Usually, this characteristic relates to how easy the insertion of these setups into the potential users’ facilities is or how workers benefit from it amidst each patient consultation. On some level, this effortlessness of inserting these setups into healthcare facilities is more important than workers’ ability to know how to do it. We argue that possible users should invest in training a group of workers and detach them for this job, given that if every worker starts doing it, it will increase the probability of someone not following the guidelines correctly, thus increasing the infection probability.

The last common advantage UVGI has compared to chemical disinfection methods is the reduced/no chemical byproduct left in SUPPE after sterilization rounds. Some studies [7,40,44,48,49,62,64,67,70,71,79,82,83] discuss this advantage. On the one hand, a few studies [7,40,83] only mention this advantage without testing it—three [70,71,82] publications tangentially discuss this characteristic by the possibility of the lasting odors resulting from the UV-C reprocessing. On the other hand, other publications [44,48,49,62,64,67,79] test for this chemical byproduct. Using low-pressure mercury lamps during the sterilization may create Ozone (O_3_), which “can pose an additional health hazard” [64] (p. 7592); if trapped inside the container, the reprocessing is taking place. Three research papers [44,48,64] find low accumulation levels of O_3_ ranging from less than 0.001 to 0.02 ppm after the UV-C sterilization process.

Still on chemical byproducts, two other publications [62,79] find some unique peaks in their analysis, but these results indicate divergent observations. Jung et al. explain that the byproducts are a result of “surface oxidation leaving some peaks of C–O–C and O–H bending” [62] (p. 11). In contrast, Salter et al. argue that their unique peaks “appear to be (…) related to the solvent (*n*-pentane) and unrelated to the disinfectant” [79] (p. 443).

Despite these advantages, there is no universal option concerning sterilization methods since all of them present disadvantages; thus, we should observe which disadvantages are present in our SLR database and if they have made this choice of reprocessing procedure inadvisable.

## 5. UV-C’s Disadvantages

There are three common problems the UV-C sterilization process demonstrates: the possibility of thermal deformation, shadowing, and absorption effects [4,5,8,27,31]. As the first potential problem (changes in integrity) already discards reprocessed SUPPE, we opted to leave it on Table 1 column “Changes in integrity or fit.” From our SLR database, 30 studies (51.72%) assess it, of which 21 [33,35,37,40,44,48,49,51,53,61,62,64,65,67,70,71,73,80,81,82,87] observe no physical changes within different rounds of sterilization or extenuating conditions. On the other hand, nine studies [36,38,42,47,54,75,78,84,86] find that masks or FFRs degraded, or faced changes in airflow resistance [36,47,75,78], or reached minimum acceptability levels after some rounds of reprocessing [38,42,54,84,86]. These results are important to consider, albeit with caution because one publication [78] indicates that despite having degradation problems, they varied according to the different models used, suggesting that it is wise to observe each case individually. In contrast, another study [75] indicates a positive relationship between degradation levels and dosage.

Another potential problem reprocessed masks and FFRs might present the reduction of their filtration power. Most studies [35,36,38,39,42,43,44,48,49,51,54,59,61,62,64,67,73,76,80,81,82,84,87] indicate that little or no effect happened as these SUPPE faced UV-C sterilization. However, this is not a consensus in the SLR’s final sample. Few publications [7,33,75,77] indicate problems in these SUPPE’s filtration power after sterilization, normally after some reprocessing cycles or in higher doses.

A third setback for choosing UV-C’s method is shadowing. This problem happens when parts of the masks or FFRs are poorly irradiated or not irradiated at all. Such a concern is a priority, especially when the object possesses inner-layers where microorganisms can remain. This problem automatically impacts UV-C’s germicidal capability because all parts must be irradiated to be decontaminated and reused. Shadowing is also a problem in these SUPPE’s straps. Some studies [1,37,40,41,44,55,58,66,67,72] discussed shadowing although only few [41,44,55,66,72] presented possible solutions. One study [41] is concerned with this problem regarding masks and FFRs straps, then to solve it, they include a fused quartz hook that enables UV irradiation. Other researchers [44,55,66,72] suggest changing the UV-C system setup or the SUPPE’s positions to increase exposure or the system’s reflection.

A fourth problem concerns UV-C’s penetration ability. This problem is intimately related to the irradiation of inner parts and with the material these SUPPEs use. Some studies [1,6,34,40,76] argue about it. The leading cause for this concern lies in the physio–chemical properties of the materials used in masks and FFRs [6,34,76]. None of these studies discussing absorption problems tried to solve them. Only one [40] argued about the possibility of optimizing their system’s setup to cope with it. Nevertheless, an increase in dosage to reach deeper layers may lead to photooxidation on the surface [42,67,75]. Thus, better reflective setups and more uniform irradiation might prove to be better solutions to reach the inner layers.

A fifth problem lies outside the capability of UV-C but in the potential users’ ability to explain to the users of reprocessed SUPPE the procedure’s safety. Only two studies [42,73] discuss it, but a system where users of the reprocessed masks and FFRs only wear their previously used SUPPE may increase acceptability.

Finally, we summarize all these potential hindrances in one. If researchers aimed at solving it, UV-C may turn into the universal method. How to improve its germicidal capability on small particles deep within masks and FFRs’ inner layers? This problem combines all possible disadvantages since it evaluates its germicidal ability, the possibility of material shadows shield these tiny particles, and the material absorption that could impede the appropriate dosage reaching them. Dealing with this problem could create more acceptance of the method in its users, and then they could use any reprocessed mask/FFR.

## 6. Conclusions

During a crisis in SUPPE, the ability of supply chains to meet the increase in demand, reprocessing these pieces of protective equipment, such as masks and FFRs, gains visibility. This visibility incentivizes academia to develop, evaluate and create multiple alternatives to sterilize them. This study aimed to describe the state-of-the-art for UV-C sterilization in masks and filtering facepiece respirators.

We used an SLR to gather information about UV-C’s germicidal capability, other advantages, and potential disadvantages. The germicidal ability combined with other benefits increases UV-C’s appeal compared to other existing sterilization methods.

Regarding the potential problems, we must consider that masks degrade in different ways [78] once they use other materials and possess individual physio–chemical properties. This consideration indicates that each model might present specific changes after UV-C sterilization rounds, and the same happens for shadowing or absorption effects [1,6]. From our SLR, we synthesize the most critical barrier for implementing UV-C sterilization as a disinfection method for masks and FFRs: How to improve its germicidal capability on small particles deep within masks and FFRs’ inner layers?

Our study might have regarded our database as another potential problem, as this topic gained relative importance after the COVID-19 pandemic. This importance increased the number of published studies significantly. Thus, we can only assert these results up to the end of January 2021, as other studies might appear after this one, and they could create new perspectives on this topic.

Therefore, a comprehensive study with multiple mask (and FFR) models, like Mills et al. [1], increases the likelihood of selecting the appropriate model(s) for UV-C sterilization, clearly explaining why the other models should not use it. Another potential avenue for future research is evaluating the physio–chemical changes masks and FFRs might pass when reprocessed by UV-C, such as the levels of chemical byproducts. Finally, UV-LEDs might be useful because they are adjustable into different shapes than the longitudinal bulbs.

## Figures and Tables

**Table 1:** SLR’s final database main features and results.

Sample	Biological Indicators	Nº of Lamps	UV-C Total Dose (J/cm^2^)	Lamp Power (W)	Exposure to UV-C (min)	Sterilization Cycles	Log Reduction	Toxic Byproduct	Filtration Powers	Changes in Integrity or Fit	Source
**VIRUSES**											
**Surgical Masks/ Procedure Masks/FFP1**											
Surgical masks ^$^	PRCV strain 91V44	4	2.6	5.5	2	1	Yes, >5	No	No	No	[34]
Surgical mask	H1N1 Influenza A virus	2	1.35	30	15	Up to 30	Yes, ≥4	No	Yes, little or no effect.	Yes, no physical changes after 30 cycles.	[35]
Surgical mask ^$^	Infectious porcine respiratory coronavirus (PRCV strain 91V44) and murine norovirus (MuNoV line RAW264.7 ATCC TIB-71)	4	2.6 ^&^	5.5	2	Up to 5	Yes, 5.37 (PRCV) and 4.65 (MuNoV)	No	Yes, little or no effect.	Yes, slightly decreased airflow resistance.	[36]
**FFP2/KN95/N95 FFR**											
N95 ^$^	H1N1 influenza (VR-1469) covered with artificial saliva or skin oil	8	1	0.39	1, 10 ^#^	1	Yes, ≥3 in 12/15 FFRs and 7/15 straps for both soiling conditions	No	No	No	[1]
N95	MS2 coliphage	1	38 to 4707	40	2 to 266	-	Yes, >3 (after 1000 J of irradiation)	No	No	No	[6]
N95	MS2 bacteriophages and Phi6	2	-	-	22 (each cycle) or 31 (once extended)	Up to 3	Yes, ≥2.1 (MS2) single cycle. >6 (three consecutive cycles or extended)	No	No	Yes, no physical changes after three cycles.	[37]
N95	Hcov-19 ncov-WA1-2020 (MN985325.1)	1	0.33 to 1.98	-	Multiple (10, 30, and 60)	Up to 3	Yes, >3	No	Yes, little or no effect.	Yes, it reached the minimum at fit test after three cycles.	[38]
N95	RIX4414 strain of the human rotavirus G1P[8] Wa strain	1	-	-	15 ^&^	Up to 5	Yes, “the rotaviral RNA was detected on both decontamination methods, while the back of N95 respirators, the rotaviral RNA was undetected.” (p. 50)	No	Yes, little or no effect.	No	[39]
N95	H1N1 influenza (VR-1469) using droplet and aerosol applications	1	1.8	80	15	1	Yes, >4 (all models both applications)	No	No	Yes, no physical changes.	[40]
N95 ^$^	Escherichia virus MS2.	8	>2	-	1	1	Yes, >3	No	No	No	[41]
N95 ^$^	Escherichia virus MS2 (MS2), Pseudomonas virus phi6 (Phi6)	8	>2	-	1	1	Yes, >2 (MS2 and Phi6)	No	No	No	[41]
N95	Vesicular stomatitis virus		Up to 1.12	-	-	Up to 5	Yes, >4	No	Yes, little or no effect after ten cycles.	Yes, effect after ten cycles.	[42]
N95	Influenza A/H5N1 (VNH5N1)	2	18,000	-	15	1	Yes, >4 (all models)	No	Yes, little or no effect.	No	[43]
KN95 ^$^	PRCV strain 91V44	4	5.2	5.5	4	1	Yes, >4	No	No	No	[34]
N95	Lentivirus bearing a GFP reporter (a surrogate for SARS-CoV-2)	-	1.8	-	<30 (7 white cycle, 10.5 colored cycle, 12 heat)	3	Yes, ~5 (UV-C alone)	Yes, minimal ozone accumulation	Yes, little or no effect.	Yes, no physical changes.	[44] *
N95	SARS-CoV-2 (USA-WA1/202)	-	-	-	Up to 5	1	Yes, >4.79	No	No	No	[45]
N95	Swine coronavirus (PEDV strain CO2013)	-	0.36 to 2.52 ^&^	25	Multiple (1, 3, 5, 7) ^&^	Multiple	Yes, 4	No	No	No	[46]
N95	Clinical samples of SARS-CoV-2	1	-	30	-	1	Yes, it depended on the model	No	No	Yes, internal degradation, producing particulate.	[47]
N95	*Staphylococcal bacteriophages* (vb_hsa_2002 and P66 phages)	10	Multiple	-	4	1	Yes, >3	Yes, minimal ozone concentration	Yes, little or no effect. (Effect caused by wearing)	Yes, no physical changes even in dosage corresponding to 50 cycles.	[48] *
N95 (one model with a hydrophilic outer layer and another with a hydrophobic outer layer)	MS2 bacteriophage (ATCC 15597-B1) (multiple deposition methods: droplets, vaporized, and aerosolized)	1	~1	40	5	1	Yes, >5 (for all models and methods).	Yes, no toxic byproduct left.	Yes, little or no effect.	Yes, no physical changes.	[49]
N95	MS2 coliphage	1	4.32	40	Up to 300	1	Yes, >3 after three hours. No virus after five hours at ~7.20 J/cm^2^.	No	No	No	[50]
N95/KN95	MS2, Phi6, influenza A virus, murine hepatitis virus	1	-	-	15	1	Yes, but <2 (MS2, Phi6, influenza A, MHV) (only UV-PX).	No	Yes, little or no effect.	Yes, no physical changes.	[51] *
N95	MS2 bacteriophage (multiple deposition methods and humidity levels)	1	-	4	Up to 240	Multiple	Yes, multiple results depending on the relative humidity of the coupon face and the deposition method (the highest was 5.8)	No	No	No	[52]
N95	Swine coronavirus (PEDV)	1	-	4	Multiple (10, 15, and 20), 1 ^#^	1	Yes, “it is likely that 10-min UV-C is sufficient for the inactivation of the virus” (p. 06)	No	No	Yes, no physical changes.	[53]
N95	Hcov-19 ncov-WA1-2020 (MN985325.1)	1	0.33 to 1.98	-	Multiple (10, 30, and 60)	Up to 3	Yes, >3	No	Yes, little or no effect after three cycles.	Yes, minimum at fit test after three cycles.	[54]
N95 ^$^	SARS-CoV-2	UV LEDs	0.3 to 0.6	-	Multiple (0, 5, and 10)	Up to 3	Yes, >3 (in one model)	No	No	No	[55]
KN95 ^$^	Infectious porcine respiratory coronavirus (PRCV strain 91V44) and murine norovirus (MuNoV line RAW264.7 ATCC TIB-71)	4	2.6 ^&^	5.5	2	5	Yes, 4.48 (PRCV) and 4.33 (MuNoV)	No	Yes, little or no effect.	Yes, no physical changes.	[36]
N95 ^$^	SARS-CoV-2 (USA-WA1/2020 NR-52281)	-	1.5 ^&^	-	1 to 1.16 ^&^	1	Yes, it depended on the model	No	No	No	[56]
N95	SARS-CoV-2 (USA-WA1/202, bei resource NR52281)	2	1.5	-	0 to 2.73	1	Yes, 3.5	No	No	No	[57]
N95	Human coronavirus NL63	1	-	-	15	1	Yes, >3	No	No	No	[58]
**BACTERIA**											
**Surgical Masks/ Procedure Masks/FFP1**											
FFP1 and surgical mask	*E. Coli* (K12) and *B. Subtilis* (B 4056)	1	Up to 0.378	-	Multiple (5, 10, 15)	1	-	No	Yes, little or no effect.	No	[59]
surgical mask	*S. Aureus*	2	1.35	30	15	Up to 30	Yes, ≥4	No	Yes, little or no effect.	Yes, no physical changes after 30 cycles.	[35]
FFP1 and surgical mask	*S. Aureus*	24	2.7	95	30	Up to 3	Yes, ≥8	No	No	No	[60]
surgical mask	*S. Aureus*	1	-	20	5 ^&^	Up to 3	Yes, 4	No	Yes, little or no effect after three cycles.	Yes, no physical changes after a 90-min exposure.	[61]
**FFP2/KN95/N95 FFR**											
N95	Methicillin-resistant *S. Aureus* (MRSA)	2	-	-	22 (each cycle) or 31 (once extended)	Up to 3	Yes, >6 (single cycle, consecutive cycles, and extended)	No	No	Yes, no physical changes after three cycles.	[37]
FFP2	*E. Coli* (K12) and *B. Subtilis* (B 4056)	1	Up to 0.378		Multiple (5, 10, 15)	1	Yes, “No surviving bacterium was observed after UVI treatment for 5 min or longer” (p. 13166)	No	Yes, little or no effect even when 20 J/cm^2^.	No	[59]
N95 (5 layers: coverweb, stiffener, 1st and 2nd filter layers, innerweb)/KF94 (3 layers: coverweb, filter web, inner web)	*E. Coli* (KCTC 1039)	1	-	10	60 ^&^	1	Yes, <2	Yes, peaks of C–O–C and O–H bending	Yes, little or no effect.	Yes, no physical changes	[62]
N95 ^$^	Methicillin-resistant *S. Aureus* (MRSA) and *C. Difficile*	8	>2	-	Up to 3	1	Yes, >5 (MRSA), <3 (*C. difficile*)	No	No	No	[41]
N95	*B. Subtilis (CCRC 12145)*	1	-	6	Multiple (1, 2, 5, 10, 20) ^&^	1	Yes, “no colony was recovered after exposure to UVC for as little as five minutes” (p. 757)	No	No	No	[63]
N95	*S. Epidermis*, *P. Aeruginosa,* and *G. Stearothermophilus*	-	1.8	-	<30 (7 white cycle, 10.5 colored cycle, 12 heat)	3	Yes, 6 (*S. Epidermis* and *P. Aeruginosa*) and > 6 (*G. Stearothermophilus*) just using UV-C	Yes, minimal ozone accumulation	Yes, little or no effect.	Yes, no physical changes	[44] *
N95	*B. Pumilus* PM-106 (as a surrogate for SARS-CoV-2)	Variable	≥1	30 (nonozone)	5, 10 ^#^	Up to 5	Yes, 6	Yes, minimal ozone concentration.	Yes, little or no effect. (one model)	Yes, no physical changes through five cycles. (One model)	[64]
N95/KN95	*E. Coli*, *S. Aureus*, and *G. Stearothermophilus*	1	-	-	15	1	Yes, but <1 (*S. aureus*) (UV-PX alone).	No	Yes, little or no effect.	Yes, no physical changes.	[51] *
N95/KN95	*S. Aureus*	24	2.7	95	30	Up to 3	Yes, ≥7	No	No	No	[60]
**FFP3/KN98/N98 FFR**											
FFP3	*E. Coli* (K12) and *B. Subtilis* (B 4056)	1	Up to 0.378	-	Multiple (5, 10, 15)	1	-	No	Yes, little or no effect.	No	[59]
**Others**											
One mask (with HEPA filter)	*B. Atrophaeus* (ATCC9372)	10	1	17	15	Up to 3	Yes, “UVC radiation eliminates pathogens in all layers ofthe HEPA filter.” (p. 13)	No	No	Yes, no physical changes.	[65]
**NO BIOLOGICAL INDICATORS/OTHER BIOLOGICAL INDICATORS**											
**Surgical Masks/ Procedure Masks/FFP1**											
Surgical mask	-	1	≥1	0.017	1	1	Yes, >5.5 (in 52 min, simulation)	No	No	No	[66] *
Surgical mask (two outers of cellulose acetate and interior of polypropylene)	-	4	1 to 10	120	~2	-	No	Yes, no toxic byproduct left.	Yes, little or no effect.	Yes, no physical changes.	[67]
surgical mask	-	2	2.7	-	5	-	No	No	No	No	[68]
**FFP2/KN95/N95 FFR**											
N95 ^$^	-	2	120 to 950	15	-	1	No	No	Yes, efficiency reduction of 1.25% in higher doses.	No	[7]
N95/KN95/KF94 ^$^	-	1	~3.6	8	30, 10 ^#^	10	No	No	No	Yes, no physical changes.	[33]
FFP2	-	3	0.3 to 3	4.9	0.183 to 100	-	No	No	No	No	[69]
N95	-	1	≥1	0.017	1	1	Yes, >5.5 (in 52 min, simulation)	No	No	No	[66] *
N95/FFP2	-	1	-	40	45	1	No	Yes, no odor.	No	Yes, no physical changes.	[70]
N95	-	1	3.24 (1.62 ^&^)	40	15	Up to 3	No	Yes, no odor.	No	Yes, no physical changes.	[71]
N95	-	Variable	0.3	-	19.4 ^&^	1	No	No	No	No	[72]
N95 (in different sizes)	-	>1	-	-	12.5 ^&^	2	No	No	Yes, little or no effect.	Yes, no physical changes.	[73]
N95	-	1	0.06	-	15 to 20	Up to 5	No	No	No	No	[74] *
N95	-	2	Multiple (1, 7, 13, 19, 31)	38	Multiple (5, 35, 65, 95, 155)	Up to 5	No	No	Yes, it presented a decrease in fiber filtration power.	Yes, it showed degradation with the increase in dosage.	[75]
N95	-	-	~1	-	5	10	No	No	Yes, little or no effect up to 10 cycles.	No	[76] **
N95	-	-	-	-	10 ^&^	1 (equivalent to 10×)	No	No	Yes, some decrease after the ninth cycle.	No	[77]
N95	-	8	-	18	10	1	No	No	No	Yes, it showed degradation after reprocessing, but levels are dependent on the model.	[78]
N95	-	-	2.7	8	60	-	No	Yes, unique peaks, but related to the *n*-pentane (solvent).	No	No	[79]
N95	-	2	-	40	Up to 480 (240 ^&^)	1	No	No	Yes, little or no effect.	Yes, no physical changes.	[80]
N95	-	1	0.176 to 0.181 ^&^	40	30 (15 ^&^)	1	No	No	Yes, little or no effect.	Yes, no physical changes.	[81]
N95^3^	-	1	-	40	30 (15 ^&^), Overnight ^#^	Up to 5	No	Yes, no odor.	Yes, little or no effect.	Yes, no physical changes.	[82]
N95/KN95	fungus *Aspergillus niger*	1	-	-	15	1	Yes, but <0.3 (UV-PX alone).	No	Yes, little or no effect.	Yes, no physical changes.	[51] *
N95 (One model with the second layer: polyester, while the other possesses a plastic-mesh in the outer layer)	-	4	1 to 10	120	~2	-	No	Yes, no toxic byproduct left.	Yes, little or no effect.	Yes, no physical changes.	[67]
N95	-	1	1	0.0001	62 to 258 ^&^	1	No	No	No	No	[83]
N95	-	4	≥1	-	4	Multiple (1, 3, 5, and 10)	No	No	Yes, little or no effect (<1.5% at 0.3 μm).	Yes, it “induced slight dose-dependent photochemical damage” (p. 03) after three cycles (p. 30).	[84]
N95	-	16	0.18 to 1.2	0.016	5	Up to 5.	No	No	No	No	[85]
N95/KN95 ^$^	-	-	-	8	30, 10 ^#^	Up to 10	No	No	No	Yes, effect after ten cycles.	[86]
N95	-	2	2.7	-	5	-	No	No	No	No	[68]
**FFP3/KN98/N98 FFR**											
FFP3	-	3	0.3 to 3	4.9	0.183 to 100	-	No	No	No	No	[69]
P3	-	1	-	40	120	1	No	No	Yes, little or no effect.	Yes, no physical changes.	[87]
**Others**											
Meltblown fabric (20 g/m^2^)	-	1	~3.6	8	30, 10 ^#^	10	No	No	Yes, reduction to 93% after 20 cycles.	No	[33]
P100	-	2	-	40	Up to 480 (240 ^&^)	1	No	No	Yes, little or no effect, but its results were more variable when the exposure period increased.	Yes, no physical changes.	[80]
P100	-	1	0.176 to 0.181 ^&^	40	30 (15 ^&^)	1	No	No	Yes, little or no effect.	Yes, no physical changes.	[81]

Observations: Grey background indicates “grey” literature, explained in section Methods. “^$^” indicates that researchers also evaluated straps. “*” indicates that authors normally considered hybrid methods instead of only UV-C. “**” indicates that there were in these authors’ sample “alternative face mask and respirator materials”, but they did not evaluate these masks after UVGI sterilization. “-” implies that the source did not provide such information. “~” signs for the idea of approximately. “^&^” indicates “(per) each side”. “^#^” stands for ambient conditions.

## Data Availability

Not applicable.

## Supplementary Materials

The following are available online at https://www.mdpi.com/2073-4360/13/5/801/s1, Figure S1: SLR’s database description, Figure S2: SLR’s database descriptive analysis. (**a**) Publications (Scholarly literature x “Grey” literature) before and after COVID-19 outbreak; (**b**) Journals’ quartiles according to SJR (2021). Observations: We suppressed the “Q4” column as there were no publications at it. “N/A” condensates studies published either in “grey” literature or in journals that were not in SJR (2021). (**c**) Journals’ area. Observations: “Health” encompasses multiples journal’s areas (“Applied Microbiology and Biotechnology,” “Medicine (miscellaneous),” “Public Health, Environmental and Occupational Health,” “Infectious Diseases,” “Ophthalmology,” “Health, Toxicology and Mutagenesis,” and “Neurology (clinical)”). “Engineering” condensates journals with the following areas: “Material Sciences (miscellaneous),” “Polymers and Plastics,” and “Engineering (miscellaneous).” “N/A” condensates “grey” literature and some journals that do not have defined areas in SJR, Table S1: Quantitative of analyzed studies and their sources.
