# Supplementary material for: Ultraviolet-C as a Viable Reprocessing Method for Disposable Masks and Filtering Facepiece Respirators
Source: Polymers (Basel). 2021 Mar 5;13(5):801. doi: 10.3390/polym13050801 (PMC7961913; doi:10.3390/polym13050801)
Supplement: Supplementary file 1 [file polymers-13-00801-s001.pdf]

polymers

MDPI

Review

Ultraviolet-C as a Viable Reprocessing Method for Disposable Masks and Filtering Facepiece Respirators

Talita Nicolau [1], Núbio Gomes Filho [2] and Andrea Zille [1,*]

[1] 2C2T - Centre for Textile Science and Technology, University of Minho, Guimarães, 4800-058, Portugal; tali_nicolau@hotmail.com
[2] School of Economics and Management, University of Minho, Braga, 4710 – 57, Portugal; id7657@alunos.uminho.pt
* Correspondence: azille@2c2t.umimho.pt

Abstract: In normal conditions, discarding single-use personal protective equipment after use is the rule for its users due to the possibility of being infected, particularly for masks and filtering facepiece respirators. When the demand for these protective tools is not satisfied by the companies supplying them, a scenario of shortages occurs, and new strategies must arise. One possible approach regards the disinfection of these pieces of equipment, but there are multiple methods. Analyzing these methods, Ultraviolet-C (UV-C) becomes an exciting option, given its germicidal capability. This paper aims to describe the state-of-the-art for UV-C sterilization in masks and filtering facepiece respirators. To achieve this goal, we adopted a systematic literature review in multiple databases added to a snowball method to make our sample as robust as possible and encompass a more significant number of studies. We found that UV-C's germicidal capability is just as good as other sterilization methods. Combining this characteristic with other advantages makes UV-C sterilization desirable compared to other methods, despite its possible disadvantages.

Keywords: Ultraviolet-C; surgical masks; filtering facepiece respirators; sterilization; germicidal capability; additional advantages; thermal deformation; shadowing; absorption effect; filtration power

Citation: Lastname, F.; Lastname, F.; Lastname, F. Title. *Polymers* **2021**, *13*, 801. https://doi.org/10.3390/polym13050801

Academic EditorChi-wai Kan
Received: 17 February 2021
Accepted: 1 March 2021
Published: 5 March 2021

Publisher's Note: MDPI stays neutral with regard to jurisdictional claims in published maps and institutional affiliations.

Copyright: © 2021 by the authors. Licensee MDPI, Basel, Switzerland. This article is an open access article distributed under the terms and conditions of the Creative Commons Attribution (CC BY) license (http://creativecommons.org/licenses/by/4.0/).

SUPPORTING MATERIAL

Materials and Methods

This paper aimed to describe the state-of-the-art for UV-C sterilization in masks and filtering facepiece respirators. We used a systematic literature review (SLR) on multiple databases to gather English-written publications that researched UV-C's impacts on masks and FFRs. The SLR did not impose yearly restrictions, and it searched in various databases: Web of Science, Scopus, PubMed, MedRxiv, bioRxiv, and Google Scholar. Besides the obvious publications, we also included in the SLR's database publications using only the raw material for masks or FFRs.

Studies that did not pass through a peer-review process are "grey" literature, like the ones available at MedRxiv, Research Square, Preprints, or a Report from Nebraska Hospital. Finally, we expanded our database using a Snowball Method to collect references that might prove useful in this context, but searched terms missed them. As this topic gained special attention after COVID-19 pandemics, this is a valuable contingency plan due to the number of publications that appeared. Table 1S summarizes the quantitative research and their respective sources in the SLR's final sample.

Table 1. Quantitative of analyzed studies and their sources.

Searched terms[1]	Web of Science[$]	Scopus	PubMed	Google Scholar[+]	medRxiv[&]	Snowball method	Total
"UV-C" +" mask*"	1	0	0	10	1	2	14
"UV-C" + "respirator*"	3	4	1	25	1	6	40
"UV-C" + "PPE"	0	0	0	3	1	0	4

[1] We suppressed the column for bioRxiv because it had no publication fitting our research parameters. "*" on the Searched Terms column represents a wildcard placed where "s" would be for collecting the plural of that term. "[$]" indicates a search on Title, Publication Name, and Topic. "[+]" indicates that we excluded citations and patents "[&]" means that we selected on these databases to match all words either on abstract or title.

To reach the SLR's final sample database with 58 references, we performed some rounds of exclusion. Initially, it accumulated 4,533 studies. In the first round of exclusion, we excluded studies duplicated or not in English and those incompatible in title, abstract, and keywords (this last one, when applicable). At this phase, we deducted 4,247 studies, remaining 286 publications in the sample. The second round of exclusion assessed the text compatibility, where we excluded 236 publications. After these two rounds, the SLR's database remained with 50 publications. After reading these publications, eight were added to the sample, as they connected to our research objective. Therefore, the SLR's final sample remained with 58 publications. Figure 1S depicts the SLR's number of publications in each.

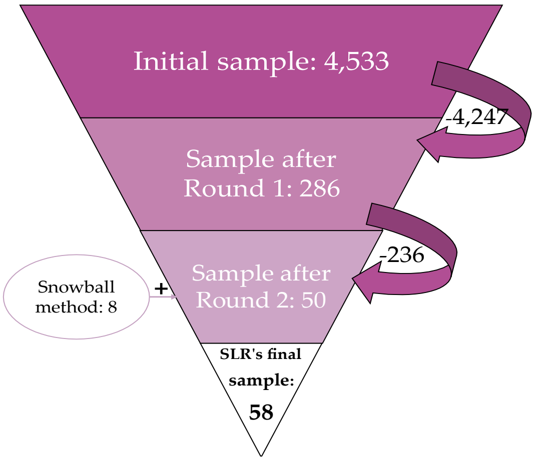

Figure S1. SLR's database description.

The SLR's final sample passed through some descriptive analysis, and Figure 2S summarizes it. Panel (a) divides the publications in terms of scholarly and "grey" literature and of date (before and after COVID-19). In this Panel, it is possible to observe that 16 studies are "grey" (27.59%), which is already a number higher than the scholarly publications before COVID-19. Readers must hold this information with caution as these studies might be going through the publication process in traditional (scholarly) journals, indicating that at some point, these numbers may shift.

Panel (b) presents the quartiles in 2019 for each Journal these studies were published, according to Scimago Journal & Country Rank (SJR). This analysis is a proxy way to define the importance of the topic, as we can observe that almost half of the studies (43.10%) were published in Q1 journals, indicating the relevance of such a subject. However, this approach is limited as some journals (nine, 15.52%) are not included at this rank, inflating the N/A group, which already agglomerates the "grey" literature.

Finally, Panel (c) indicates the Journal's area. SJR (2021) provides information about each Journal's area, which was crucial to creating them. "Health" and "Engineering" encompass multiple areas. Excluding "N/A" from the analysis, numerous papers (25,

representing 43.10% of the sample) were published at "Health" journals, while only six (10.34%) are at "Engineering" journals and two (3.45%) in "Multidisciplinary" journals. These numbers might indicate that readers looking for reprocessing masks and FFRs, and its advantages (discussed in subsections 3.1 and 3.2) after COVID-19's outbreak tend to be healthcare workers, hospitals, and academics.

On the one hand, readers must be careful, given that reprocessing masks and FFRs also appear in publications outside this scope. On the other hand, researchers must be aware of their publications' potential readers and "read the audience" while writing and analyzing data to maximize their repercussions. Researchers must also provide as much data as possible about their experiments to guarantee that their results are replicable in healthcare facilities if needed.

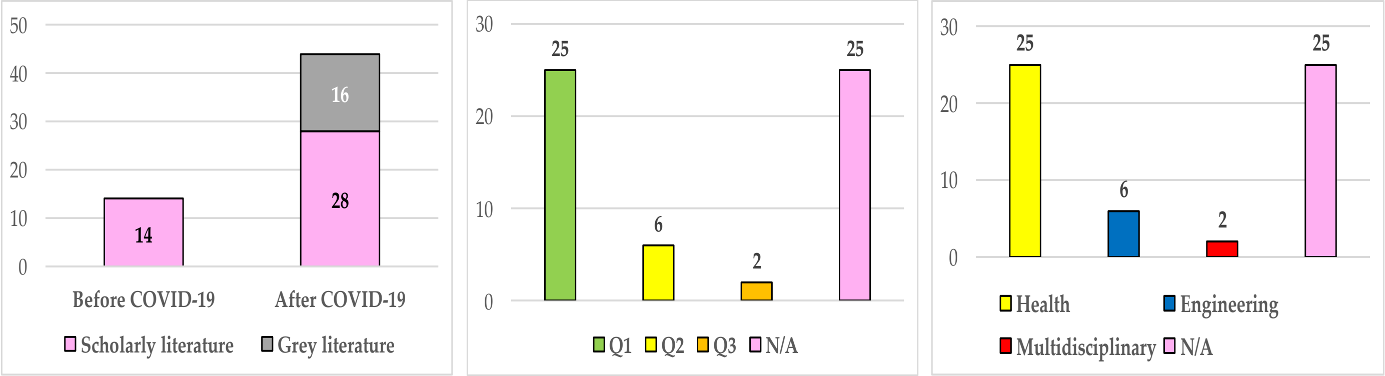

(a) (b) (c)

Figure S2. SLR's database descriptive analysis. (**a**) Publications (Scholarly literature x "Grey" literature) before and after COVID-19 outbreak; (**b**) Journals' quartiles according to SJR (2021). Observations: We suppressed the "Q4" column as there were no publications at it. "N/A" condensates studies published either in "grey" literature or in journals that were not in SJR (2021). (**c**) Journals' area. Observations: "Health" encompasses multiples journal's areas ("Applied Microbiology and Biotechnology," "Medicine (miscellaneous)," "Public Health, Environmental and Occupational Health," "Infectious Diseases," "Ophthalmology," "Health, Toxicology and Mutagenesis," and "Neurology (clinical)"). "Engineering" condensates journals with the following areas: "Material Sciences (miscellaneous)," "Polymers and Plastics," and "Engineering (miscellaneous)." "N/A" condensates "grey" literature and some journals that do not have defined areas in SJR.
